# Added value of post-mortem computed tomography (PMCT) to clinical findings for cause of death determination in adult “natural deaths”

**DOI:** 10.1007/s00414-019-02219-6

**Published:** 2019-12-18

**Authors:** M. E. M. Vester, R. R. van Rijn, W. L. J. M. Duijst, L. F. M. Beenen, M. Clerkx, R. J. Oostra

**Affiliations:** 1 Academic Medical Center Amsterdam, University of Amsterdam, Amsterdam, Netherlands; 2 Department of Radiology and Nuclear Medicine, Amsterdam UMC, University of Amsterdam, Room G2-231, Meibergdreef 9, Amsterdam, the Netherlands; 3 Department of Forensic Medical Investigations, Netherlands Forensic Institute, The Hague, the Netherlands; 4 Amsterdam Center for Forensic Science and Medicine, Amsterdam, the Netherlands; 5 Faculty of Law, Maastricht University, Maastricht, the Netherlands; 6 Department of Forensic Medicine, GGD IJsselland, Zwolle, the Netherlands; 7 Department of Medical Biology, Section Clinical Anatomy and Embryology, Amsterdam UMC, University of Amsterdam, Amsterdam, the Netherlands

**Keywords:** Autopsy, Tomography, X-ray computed, Pathology, Anatomy

## Abstract

**Purpose:**

The aim of this study was to investigate whether post-mortem computed tomography (PMCT) provides additional information regarding the cause of death and underlying diseases in a general practitioners’ (GP), out-of-hospital population.

**Methods and materials:**

Bodies donated to our anatomy department between January 2014 and January 2018, who consecutively underwent a total body PMCT and had given permission for retrieval of their medical records during life, were included. PMCT scans were assessed by a radiologist and compared with the cause of death as stated in the medical records. Discrepancies were analyzed with an adjusted Goldman classification.

**Results:**

Ninety-three out of the 274 scanned donors during the inclusion period had given consent for the retrieval of their medical records, of which 79 GP’s responded to the request thereof (31 men, 48 women, average age 72.8 years, range 36–99). PMCT identified 49 (62%) cases of cancer, 10 (12.7%) cardiovascular diseases, 8 (10.1%) severe organ failures, 5 (6.3%) cases with signs of pneumonia, 2 (2.5%) other causes, and 7 (8.9%) cases without an (underlying) definitive cause of death. Eleven major discrepancies on the Goldman classification scale, with possible relevance to survival between PMCT and GP records, were identified.

**Conclusion:**

PMCT can have added value for the detection of additional findings regarding the cause of death in an out-of-hospital, GP’s population, especially to identify or exclude major (previously non-diagnosed) underlying diseases.

## Introduction

Cause and manner (natural or non-natural) of death assessment, as assessed by the attending physician or municipal coroner, is a frequent topic of public and scientific debate [1–4]. In the Netherlands, registration and documentation on the death certificate are both legally obligatory and important for population statistics and health care quality control [5]. In natural deaths, cause of death determination is often based on medical history and circumstantial evidence rather than a post-mortem in-depth medical examination. This sometimes allures to conclusions such as dying of “old age” when the cause of death remains unknown. Furthermore, even with appropriate diagnostic tests shortly before the demise available (hospitalized patients), up to a third of cause of death diagnosed in the Netherlands are incorrect [3]. For an accurate cause of death determination, autopsy is considered the reference standard.

Clinical autopsy however is a labor-intensive, time-consuming, and often costly procedure, which in the Netherlands is not paid for by health care insurance companies. Out-of-hospital autopsies are usually more cumbersome to arrange as general practitioners (GP) have no direct access to hospital pathology services. Furthermore, already present ante-mortem imaging and laboratory results leading to a possible cause of death, or on the other hand, moral objections can hinder professionals to ask relatives for autopsy permission [6]. The invasive character of autopsy can be deterring to relatives or be in conflict with by their religion. As a result of a combination of all the aspects mentioned, the number of autopsies has been decreasing worldwide over the last decades [7–9].

In recent years, the use of post-mortem computed tomography (PMCT) as a non-invasive, relatively cheap, fast, and widely available technique has been proposed to assist in cause of death determination. This could improve health care quality control, mortality statistics, prevention programs, and possibly the detection of (unknown) hereditary diseases. In a trauma setting, PMCT has proven to be a good alternative for those cases in which autopsy is not possible [10]. Yet, PMCT as a diagnostic tool for cause of death determination has not been evaluated in out-of-hospital deaths of a GP population. If PMCT can also improve cause of death diagnosis and identification of underlying diseases in GP patients, this might be a more acceptable, non-invasive, and affordable alternative to autopsy in this population as well as a valuable tool for professional quality control. Therefore, the purpose of this study was to answer two questions. Does PMCT provide additional information regarding the cause of death and underlying diseases in a GP population? Secondly, does this PMCT-based diagnosis comply or oppose with cause of death as determined by the GP?

## Materials and methods

### Legal foundation, study design, and study population

On the basis of Article 67–69 of the Burial Act, adult inhabitants of the Netherlands can register in any of the eight Dutch medical universities, to donate their body to science and education. Since 2014, all donors of the Amsterdam UMC, University of Amsterdam, body donation program, sign an additional statement in which the donors give permission to retrieve their medical records. In addition, all living donors, registered between 1-1-2008 and 31-12-2013 (*n* = 1465), were contacted by mail in order to obtain the same permission. This request had a response rate of 1201 (82%), including four (0.003%) objections to retrieval of medical records. For this study, a waiver was issued by the medical ethical committee of our hospital.

In this case series, bodies donated to the anatomy department of the Amsterdam UMC, University of Amsterdam, arriving between January 2014 and January 2018 and who underwent a total body PMCT, were included. All donors died either due to natural causes, as reported by the GP, or in case of a non-natural cause of death, e.g., euthanasia, were released from legal custody by the public prosecutor. Cases without permission to obtain medical history were excluded from this study. GP’s were contacted to obtain the medical records by the first author (MV). Specifically, information regarding the cause of death was requested along with a summary of the medical history, medication, and known hereditary diseases. No gender, age, post-mortem interval, body mass index, or amputations/body deformity limitations were applied in this study.

### Post-mortem imaging

**Fig. 1:**
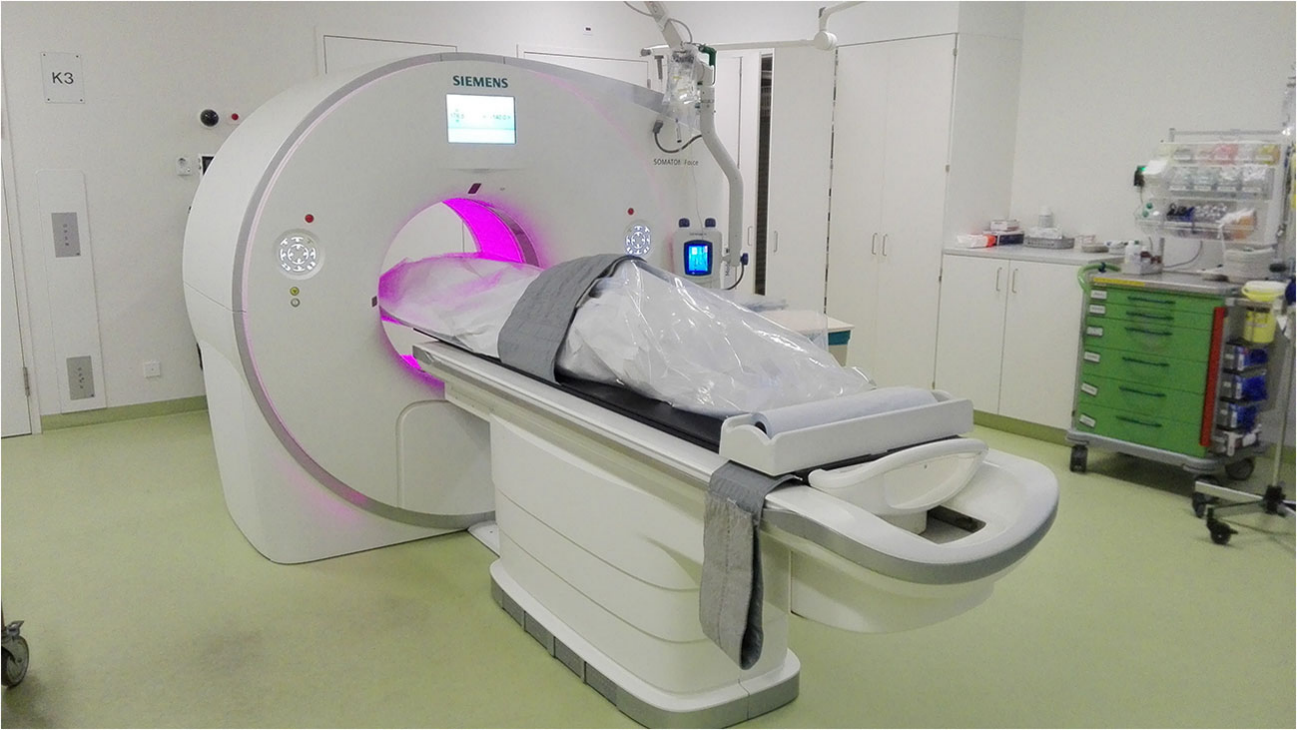
Scanning of study body in a body bag on the Siemens SOMATOM Force® Dual source CT scanner

As part of the standard workup procedure for educational purposes, a total body PMCT was made as soon as possible upon arrival at the department of anatomy, before embalming. Due to personnel or technical difficulties, PMCT scanning was not always possible. During the CT scanning procedure, the arms were crossed over the thoracoabdominal region, in a supine position within a body bag (Fig. 1). Up to June 2015, a Siemens Sensation 64® CT scanner (Siemens Healthineers, Erlangen, Germany) was used with set parameters for 3 mm slice thickness; 3 mm increments; 120 kV tube voltage; 325 mAs tube current; B30f (soft tissue filter) convolution kernel. After June 2015, a Siemens SOMATOM Force® dual source CT scanner (Siemens Healthineers, Erlangen, Germany) with dual energy capabilities became available. The dual energy scans were made at 100 kV with 750 mAs and Sn 150 kV with 375 mAs. Collimation was 2 × 192 × 0.6 mm, with axial and coronal reconstructions (1 mm Br40 and Qr40 soft tissue filters and 3 mm Br59 bone filters), along with dedicated reconstructions for dual energy post-processing. No clinical autopsy or other invasive post-mortem procedures were performed prior to embalming.

### Data analysis

A forensic radiologist with 14 years of experience, in PMCT, (RvR) assessed the scans for the cause of death, underlying diseases, and other abnormalities. These assessments were initially made without the medical records and were reviewed a second time in a different order at least half a year later with knowledge of the medical records. All findings were recorded on a standardized case report form. Findings were recorded per body location and classified into (1) tumors and/or metastases, (2) cardiovascular diseases (e.g., myocardial infarction), (3) signs of infections, (4) signs of organ failure (liver cirrhosis, severe COPD, renal failure), (5) other; not relevant to the cause of death (gallstones, old fractures, rheumatism), and (6) signs of surgery or interventions (stent, coils, prostheses, pacemakers).

Cause of death as diagnosed by PMCT was compared with what was stated in the medical records. Analysis of any discrepancies between the two were scored according to the classification of Goldman et al. [1], later adapted by Battle et al. and Schwanda-Burger et al. [11, 12]. This discrepancy classification method was originally developed for ante-mortem clinical diagnosis and post-mortem autopsy comparisons (Table 1). For this study, discrepancies were scored independently by three individuals: two radiologists (RR and LB) and one forensic physician (WD). In case of differences, a consensus was met after discussion.

**Table 1:** Classification of discrepancies between clinical records and PMCT results [1, 12]

Classification	Explanation
Class I	Major discrepancies, with prolonged survival or healing
Class II	Major discrepancies, without prolonged survival or healing
Class III	Minor discrepancies diagnosis that would have
Class IV	Minor discrepancies, diagnosis that would have had epidemiological or genetic consequences
Class V	Non-discrepant results
Class VI	Non-classifiable findings

## Results

### Subject identification

**Fig. 2:**
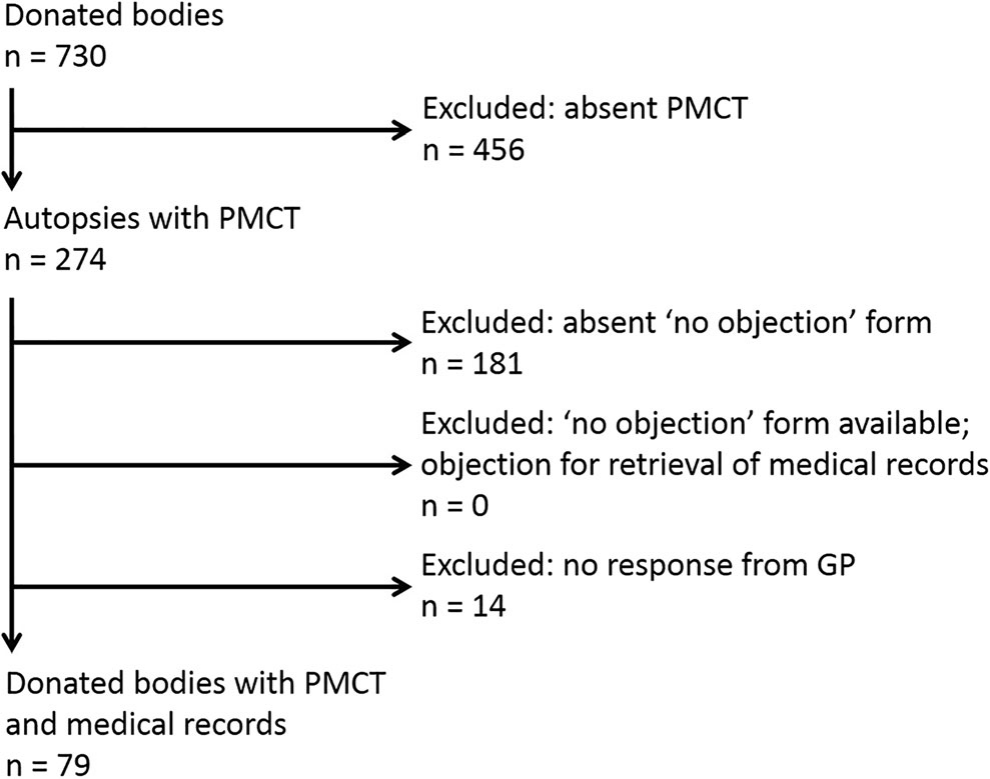
Flow diagram of selected cases donated from 1 to 1-2014 to 1-1-2018

During the four-year study period, 730 bodies were donated to our anatomy department (Fig. 2). Ninety-three out of the 274 scanned donors had given consent for the retrieval of their medical records, of which 79 GP’s responded to the request. In total 31 men and 48 women were included with an average age of 72.8 years (range 36–99 years, SD 10.1). Median time between death and arrival at the anatomy department and PMCT was 27 h (range 4–72 h, SD 17.2).

### Medical records

**Fig. 3:**
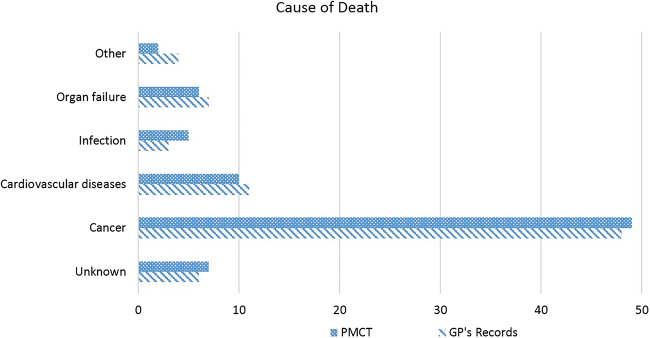
Cause of death as determined by the GP clinically and by the radiologist based on PMCT

According to the medical records provided by the GP’s, 50 cases (63.3%) received no specific last phase of life treatment, 13 cases (17.3%) died in the course of palliative care and 16 died a non-natural death including 15 cases of euthanasia (20%), and a single case of suicide (1.3%). Recorded causes of death, including grounds for palliative care or euthanasia, were mostly cancer (*n* = 48, 60.8%). Other causes were cardiovascular diseases (*n* = 11, 13.9%), organ failure (*n* = 7, 8.9%), infections (*n* = 3, 3.8%), or other/unknown (*n* = 4, 5.1%) (Fig. 3).

### Post-mortem computed tomography

Signs of palliative care of euthanasia are non-existent on PMCT and were thus not found in any of the concerning cases, nor were there signs of suicide. In 49 (62%) cases, cancer was diagnosed, in 10 (12.7%) cases severe cardiovascular diseases, in 8 (10.1%) cases signs of terminal organ failure (e.g. COPD), in 5 (6.3%) cases signs of infection (pneumonia), 2 (2.5%) other causes, and in 7 (8.9%) cases no (underlying) radiological diagnosis of the cause of death could be established (Fig. 3). Assessments on cause of death on PMCT did not alter after medical information was given to the radiologist.

### PMCT comparison with medical records

Added value of PMCT to GP records was found in 12 (15.2%) cases, consisting of 11 major discrepancies with relevance to survival (class I discrepancies) (Table 2) and 1 major discrepancy without influence to survival (class II discrepancy) (Fig. 5). Two of these class I discrepancies were subjects who died after palliative care. PMCT was also of additional value for a single case of undiagnosed and untreated osteomyelitis (minor discrepancy; classification score III). Sixteen cases (20.3%) were scored as non-classifiable (classification VI) because PMCT was unable to identify the cause of death or underlying disease, including the single suicide case. For the remaining 50 cases (63.3%), there was no discrepancy between clinical diagnosis and PMCT findings (classification score V). Discussion between the different observers was needed in 10 out of the 79 cases to reach consensus on the allocated discrepancy scores.

**Table 2:** Cases with major discrepancies with relevance to survival (Goldman classification I) between PMCT and GP records (standard care comprehends continuation of treatment without specific last phase of life treatment such as palliative care)

No	Sex	Age	GP’s diagnosis	PMCT diagnosis	Last/final care	Discr. score
1	F	69	Metastasised lung carcinoma	Metastasised lung carcinoma with signs of a pneumonia	Standard care	I
2	M	71	Unknown cause of death	Lytic bone lesions DD multiple myeloma, metastasised carcinoma (Fig. 4)	Standard care	I
3	M	79	Aspergilloma	Lesion left upper lobe and pneumonia left lower lobe with pleural empyema	Palliative care	I
4	F	69	Respiratory insufficiency due to st. IV adenocarcinoma NSCLC	Pneumonia	Standard care	I
5	F	88	Pneumonia and clostridium difficile infection	Congestive heart failure	Standard care	I
6	F	77	Probably lung carcinoma	No abnormalities at PMCT (no lung abnormalities)	Standard care	I
7	M	74	Metastasised stomach carcinoma	Metastasised carcinoma, probably pancreatic carcinoma	Standard care	I
8	F	66	Metastasised stomach carcinoma	Metastasised stomach carcinoma with untreated carcinomatous pleurisy	Standard care	I
9	F	71	Hospitalisation due to gallbladder perforation	Severe emphysema, mediastinal lymphadenopathy; infection	Standard care	I
10	M	90	Hospitalisation due to gallbladder perforation	Metastasised carcinoma	Palliative care	I
11	F	85	Myocardial infarction-atrial fibrillation	Metastasised carcinoma	Standard care	I

**Fig. 4:**
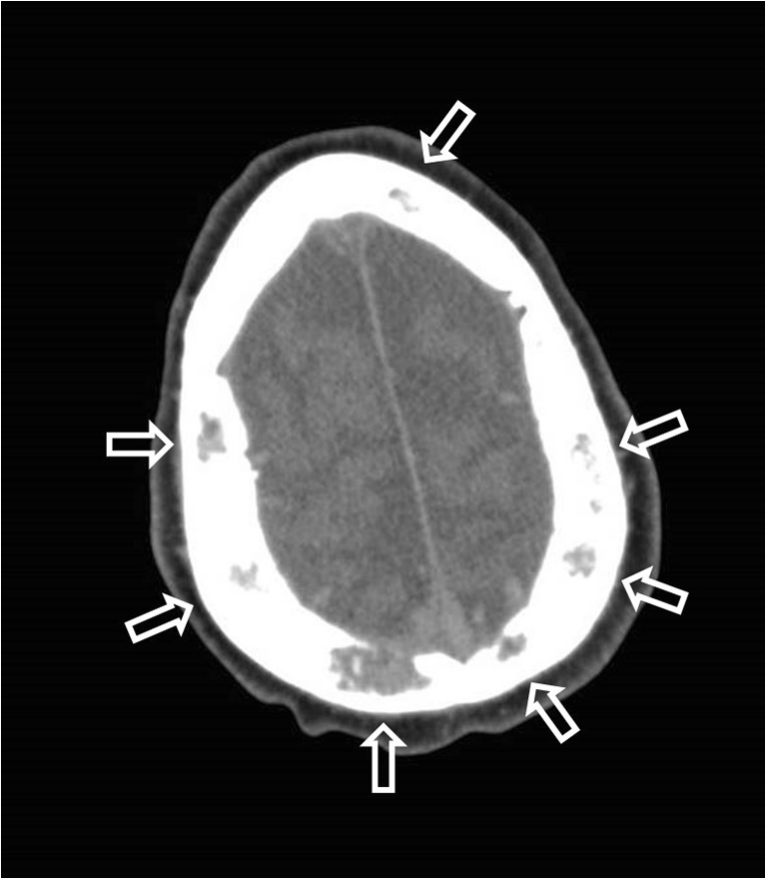
Lytic bone lesions in the cranial bones due to either metastasised carcinoma or multiple myeloma, unknown to the General Practitioner

**Fig. 5:**
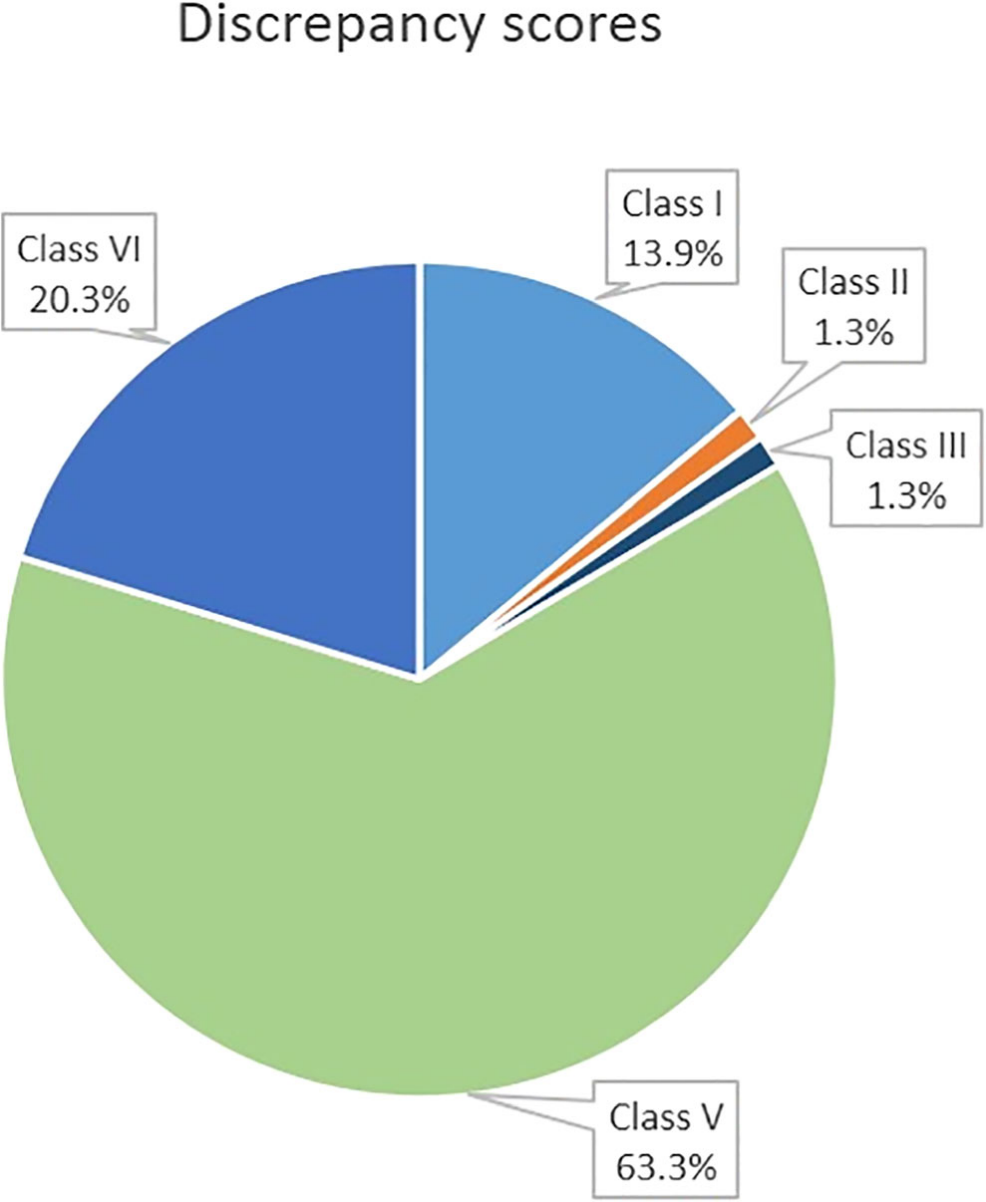
Abnormalities (potentially) relevant to the cause of death

## Discussion

Our results indicate that in the majority of cases PMCT does not provide additional information regarding the cause of death. However, in 11 out of 79 cases (13.9%), a major discrepancy was noted in diagnosis between GP documents and PMCT results, which would have required treatment and could have possibly prolonged survival. These finding imply that PMCT, though as a single modality is known to be an unreliable reference standard for cause of death determination [13, 14], does seem to be a reliable method for identification of additional information regarding the cause of death and underlying diseases in a GP’s practice population. Especially, since autopsy is generally uncommon in this group. Autopsy, as noted in the introduction, is the reference standard for cause of death determination and self-control of medicine [15]. When performed in an experienced setting and without histological examination, autopsy is a relatively quick procedure which should be always pursued if there is a question related to the cause of death. However, autopsy in general has become less common over the last decades due to, among others, its invasive character. There has been a decrease of 0.7% per year for in-hospital deaths in the Netherlands between 1977 and 2011 [16]. It has been postulated that this decrease in post-mortem examinations might cause for important complications and disorders to be missed. Consequently, this causes a decrease in accurate statistics and health care quality control [3, 16, 17].

The priority of death investigations in the Netherlands lies with differentiation between natural and non-natural deaths, with the actual cause of death secondary to that. But, post-mortem cause of death evaluation and diagnosis are, among others, essential to medical quality control and identification of previously unknown genetic diseases [18]. Moreover, epidemiological statistics form the basis of education, funding, and research are related to the cause of death determination. According to the Statistics Netherlands for instance, acute cardiovascular incidents of both ischemic and rhythmic origin are frequently assigned as the cause of death, adding up to 21.1% [19]. This results amongst others in a higher availability of research funding for cardiovascular diseases. Currently, GP’s are in most cases limited to exploration of the medical history and a physical external examination for their cause of death conclusions. Distinguishing between death due to an acute myocardial infarction, (ischemic) stroke, thromboembolism, rupture of an (unknown) aneurysm or other causes by solely the medical history and a physical external examination is possible, but can be difficult. In some cases, this is too little evidence for an accurate determination of the cause of death. Subsequently, the determination of the cause of death is based on probability, which is, among others, based on the national epidemiological statistics. Thenceforward, the cause of death documents are used to determine these statistics, hence confirming itself, causing a circle reasoning.

The next step in post-mortem imaging is contrast-enhanced PMCT (PMCT angiography; PMCTA) or PMCT combined with, e.g., toxicology or targeted biopsies (minimal invasive autopsy, MIA). In a prospective study of 210 cases, a cause of death was determined by PMCTA in 92% and was superior for trauma and hemorrhages [14], though pulmonary thromboembolism was better detected by autopsy. Overall, PMCTA had 6% major discrepancies compared with PMCTA combined with autopsy, and autopsy had 5% major discrepancies. Furthermore, the combination of PMCTA and autopsy showed superior results for the detection of abnormalities and the cause of death in 500 cases [13]. Here, autopsy, PMCT, and PMCTA individually detected 61.3%, 76.0%, and 89.9% findings, respectively. PMCT and PMCTA proved to be superior over autopsy especially for skeletal and vascular lesions. A large systematic review, in suspected natural deaths, showed that PMCT combined with MRI gives the best results of non-invasive techniques for the cause of death determination with 70% agreement with autopsy. Minimal invasive autopsy (PMCT, PMCTA, and biopsy) achieved a 90.9% agreement [20]. Nonetheless, a recent study argues that biopsies are only an alternative when consent for an autopsy cannot be obtained, since they found that CT-guided post-mortem biopsies of the lungs had a mediocre predictive value [21].

Our study has some limitations. Though the study population is representative in age distribution for the general population, it is a relatively small sample size. Secondly, death certificates would ideally be compared with clinical autopsies, macroscopic, and/or microscopic internal examination. Yet, death certificates are anonymous in the Netherlands and thus unavailable for a retrospective study, leaving the sometimes incomplete GP’s records for review. PMCT as a diagnostic tool has some important limitations as well. Even though PMCT can identify signs of a possible pneumonia, the definite diagnosis cannot be made without clinical symptoms and/or laboratory tests. Furthermore, dementia is one of the most important causes of death, yet it is a clinical diagnosis based on symptoms [22, 23], and it is thus impossible to designate this as the cause of death by PMCT. Euthanasia does not leave traces detectable by PMCT, though this does not pose a problem since it is the underlying disease vindicating the euthanasia, which is of importance for quality control and statistics. There have been multiple publications emphasizing the need for autopsy to avert missing non-natural deaths, including homicides [3, 24]. For these cases, an extensive external examination combined with PMCTA and or minimal invasive autopsy would be recommended based on literature. Nevertheless, transportation, availability, and costs are limiting factors to put this into daily practice currently when there are no clinical suspicions. Furthermore, as for any subspecialty, assessment of post-mortem imaging requires a certain level of training, knowledge, and expertise, as was the case in our study [25–27]. This subspecialty is gaining each year with dedicated training programs, but is not yet present in all clinical centers. Lastly, there was a change in the CT scanner during the study period which might have improved diagnostic accuracy of the later cases. However, this possibly improved diagnostic accuracy is unlikely to be of significant influence on the study result.

In deaths without a known recent medical history attesting to the demise, PMCT could be used to identify or exclude a major underlying disease in addition to an autopsy or when an autopsy is not possible. Future studies should therefore focus on a broader population of out-of-hospital, suspected natural deaths with the additional use of autopsy and/or PMCT(A). With funding such a study, improvement of quality control and epidemiological statistics could be obtained and possibly a redistribution of funding in the future. Thus to conclude, our study suggests that there is added value for PMCT in the identification of diseases and illnesses leading to the demise in an out of hospital patient population.

## Abbreviations

GP: General practitioner
PMCT: Post-mortem computed tomography
